# Bactericidal Performance of Visible-Light Responsive Titania Photocatalyst with Silver Nanostructures

**DOI:** 10.1371/journal.pone.0010394

**Published:** 2010-04-29

**Authors:** Ming-Show Wong, Der-Shan Sun, Hsin-Hou Chang

**Affiliations:** 1 Department of Materials Science and Engineering, National Dong-Hwa University, Hualien, Taiwan; 2 Nanotechnology Research Center, National Dong-Hwa University, Hualien, Taiwan; 3 Department of Molecular Biology and Human Genetics, Tzu-Chi University, Hualien, Taiwan, Republic of China; 4 Tzu-Chi University Center for Vascular Medicine, Tzu-Chi University, Hualien, Taiwan, Republic of China; Charité-Universitätsmedizin Berlin, Germany

## Abstract

**Background:**

Titania dioxide (TiO_2_) photocatalyst is primarily induced by ultraviolet light irradiation. Visible-light responsive anion-doped TiO_2_ photocatalysts contain higher quantum efficiency under sunlight and can be used safely in indoor settings without exposing to biohazardous ultraviolet light. The antibacterial efficiency, however, remains to be further improved.

**Methodology/Principal Findings:**

Using thermal reduction method, here we synthesized silver-nanostructures coated TiO_2_ thin films that contain a high visible-light responsive antibacterial property. Among our tested titania substrates including TiO_2_, carbon-doped TiO_2_ [TiO_2_ (C)] and nitrogen-doped TiO_2_ [TiO_2_ (N)], TiO_2_ (N) showed the best performance after silver coating. The synergistic antibacterial effect results approximately 5 log reductions of surviving bacteria of *Escherichia coli*, *Streptococcus pyogenes*, *Staphylococcus aureus* and *Acinetobacter baumannii*. Scanning electron microscope analysis indicated that crystalline silver formed unique wire-like nanostructures on TiO_2_ (N) substrates, while formed relatively straight and thicker rod-shaped precipitates on the other two titania materials.

**Conclusion/Significance:**

Our results suggested that proper forms of silver on various titania materials could further influence the bactericidal property.

## Introduction

Since the widespread use of antibiotics and the emergence of more resistant and virulent strains of microorganisms, there is an urgent need to develop alternative sterilization technologies. Traditional ultraviolet (UV) light responsive titania dioxide (TiO_2_) photocatalyst has been demonstrated to eliminate organic compounds and use as disinfectants [1]. The photon energy generates pairs of electron and hole that yield reactive oxygen species (ROS) such as hydroxyl radicals (· OH) and superoxide anions (O2^−^) that working as biocides [2]. Since TiO_2_ is a chemically inert compound, which can exert antimicrobial activity persistently and be controlled by illumination; it seems to be a conceptually feasible alternative. Because it absorbs only 2–3% UV-range of solar light impinging on the Earth's surface when used for an outdoor setting [3], and UV-light can induce damages of human cells and prohibits the use in our living environments, new generation of visible-light responsive titania photocatalysts were developed [4]. To extend the light-absorption into visible-light range thereby increasing quantum efficiency and safety, doping with transition metal ions and/or anions (negative ions) on TiO_2_ is a commonly used method, by which it creates intra-band gap states close to the conduction or valence band edges that induces visible-light absorption at the sub-band gap energies [3], [4]. The ion/anion doped materials may also inhibit the charge recombination, thereby increase the photocatalytic activity [3], [5]–[7]. Using such approach, several studies have shown to develop titania photocatalyst with antimicrobial activity in the visible-light range [6]–[17]. The drawback of visible-light titania photocatalyst, however, is the low antibacterial efficiency as compared with traditional chemical disinfectants.

Among the doping materials for the development of visible-light titania photocatalyst, silver is one of the interesting element. Silver has been used as bactericidal agent in hygiene and medicinal applications for thousands of years [18]. Currently the antibacterial property of silver is wildly used for managing and cleaning of burn, trauma and diabetic wounds, catheter, and dental silver amalgams [19], [20]. Both ions (Ag^+^) and nanoparticles of silver were shown to have antibacterial activity [18], [19], [21]. Silver ions could affect the bacterial membrane respiratory electron transport chains and DNA replication components [20]. Studies indicated that silver/TiO_2_ composites may enhance the photocatalytic destruction of *E. coli* [22]–[25]; however, the bacterial-killing enhancement of photocatalysis might not apply to all forms of silver coating [26]. These results indicated that silver/TiO_2_ composted materials may contain the advantages of both materials: silver has a higher antimicrobial activity, and TiO_2_ can last longer, and able to be controlled by illumination. The conditions of silver-coating, however, might need a defined justification. Thus, here we investigated the antibacterial performance of silver containing visible-light responsive TiO_2_ (C) and TiO_2_ (N) substrates, and the influence of silver dosage/deposition status on the bactericidal property using bacterium *E. coli*. The antibacterial activity against pathogenic bacteria *Streptococcus pyogenes*, *Staphylococcus aureus*, and *Acinetobacter baumannii* was also discussed.

## Results

### Bactericidal activities of TiO_2_ substrates coated with or without silver

**Figure 1:**
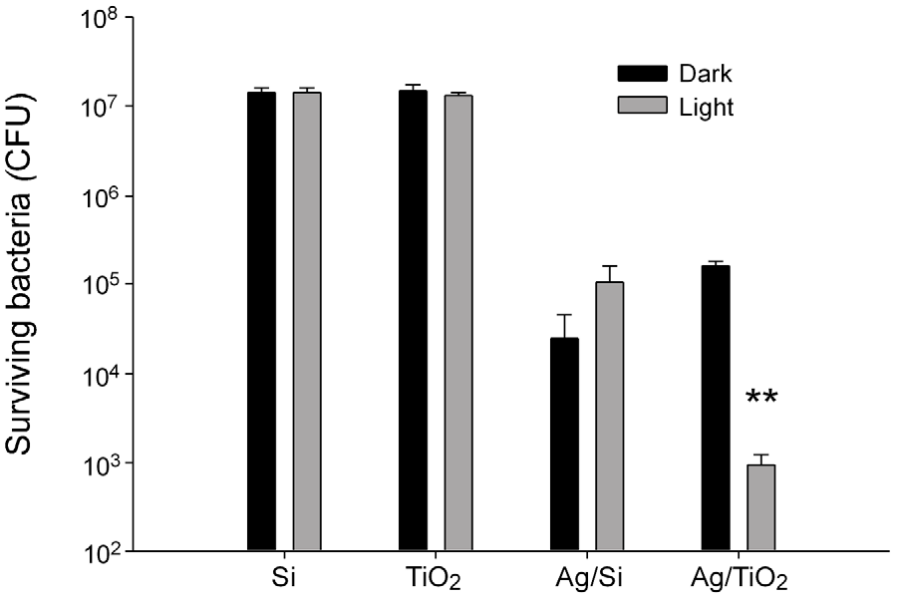
Bactericidal performance of silver containing titania photocatalyst. Antibacterial activities against *E. coli* of Ag/TiO_2_-coated silicon substratum with (Light) or without (Dark) visible light illumination were shown. ** *P*<0.01 as compared with both “Dark” and “Light” groups on Si and TiO_2_ substrates, and “Dark” groups on Ag/TiO_2_ substrates.

To investigate the bactericidal property of TiO_2_ substrate with or without silver, the survival rate of *E. coli* (1×10^7^ CFU) after visible-light-induced photocatalysis on four substrates including Si, TiO_2_, Ag/Si, and Ag/TiO_2_ were examined. We found that silver-coating alone is sufficient to exhibit a strong antibacterial activity without illumination (Fig. 1 “Dark” condition in both Ag/Si, and Ag/TiO_2_ groups). In addition, visible light illumination on Ag/TiO_2_ substrates further enhanced the bactericidal property (Fig. 1 “Light” vs. “Dark” condition in Ag/TiO_2_ groups). Notably, TiO_2_ is primarily working as an UV-light responsive photocatalyst. These results suggested that silver-depositions in this system changed the light responsiveness and the antibacterial property of TiO_2_ surfaces.

### Different silver-depositions on carbon- and nitrogen-doped TiO_2_ substrates

**Figure 2:**
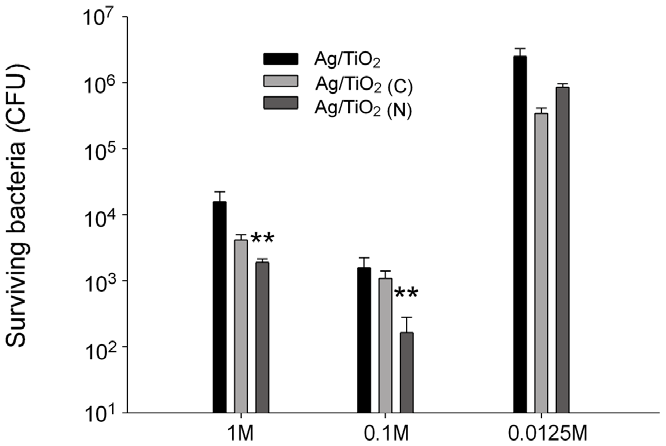
Bactericidal performance of photocatalysts with different silver preparation. TiO_2_, TiO_2_ (C) and TiO_2_ (N) substrates were coated with silver depositions by thermal reduction method using solutions containing 1 M, 0.1 M and 0.0125 M of AgNO_3_, respectively. Surviving *E. coli* (CFU) on respective substrates after photocatalysis (1.2×10^3^ lux, 10 min) was shown. ** *P*<0.01 as compared with respective Ag/TiO_2_ and Ag/TiO_2_ (C) groups.

Previously we found that dopants of carbon and nitrogen could enhance the visible-light induced antimicrobial property of TiO_2_ [13], [17], [27], here we would like to further investigate the influence of silver-depositions on carbon- and nitrogen-doped TiO_2_ substrates [TiO_2_ (C) and TiO_2_ (N)]. In addition, we also investigated whether we could control the silver deposition through changing the AgNO_3_ concentration during thermal reduction. As a result, TiO_2_, TiO_2_ (C) and TiO_2_ (N) substrates were coated with silver by thermal reduction method using different AgNO_3_ concentration. During the preparation of sliver-containing photocatalytic surfaces (300°C, 3 hours), AgNO3 decomposed completely and transform totally into pure silver. Thus, the average amount of silver depositions on the three different samples [TiO_2_, TiO_2_ (C) and TiO_2_ (N)] is the equal in groups with same AgNO_3_ concentration (data not shown). *E. coli* (1×10^7^ CFU) were then subjected to visible-light induced catalysis on these substrates. We found that dopants of nitrogen [Ag/TiO_2_ (N) groups] further enhanced the bactericidal performance of Ag/TiO_2_ (Fig. 2, ** *P*<0.01 as compared with respective Ag/TiO_2_ groups). Furthermore, 0.1 M AgNO_3_ is the best condition used herein, since either higher (1 M) or lower (0.0125 M) concentration of AgNO_3_ resulted a less performance [Fig. 2, 0.1 M vs. 1 M and 0.0125 M in Ag/TiO_2_ (N) groups].

### Kinetic analysis

**Figure 3:**
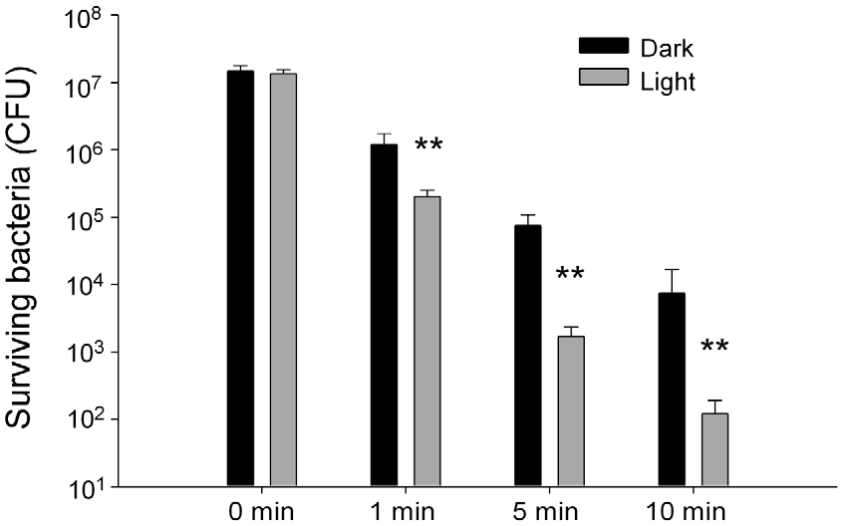
Kinetics analysis. Antibacterial activity of Ag/TiO_2_ (N) substrates was evaluated using *E. coli*. Visible-light illumination (Light) was carried out at a light density of 1.2×10^3^ lux (30 mW/cm^2^) for different time points. The silver coating is derived from thermal reduction of 0.1 M AgNO_3_. After photocatalytic treatments, the surviving bacteria (CFU) were then compared with groups without light illumination (Dark). ** *P*<0.01, as compared with both 0 min groups and respective groups without illumination (Dark).

To obtain a kinetic data, we further analyzed the *E. coli* on Ag/TiO_2_ (N) substrates (0.1 M AgNO_3_ groups in Fig. 2) that illuminated by visible light at various time points (Fig. 3). It showed that Ag/TiO_2_ (N) substrates could efficiently reduce the *E. coli* population in dark, and the exposure to various degrees of illumination could further enhance the antibacterial performance over time (Fig. 3).

### Electron microscopic analysis

**Figure 4:**
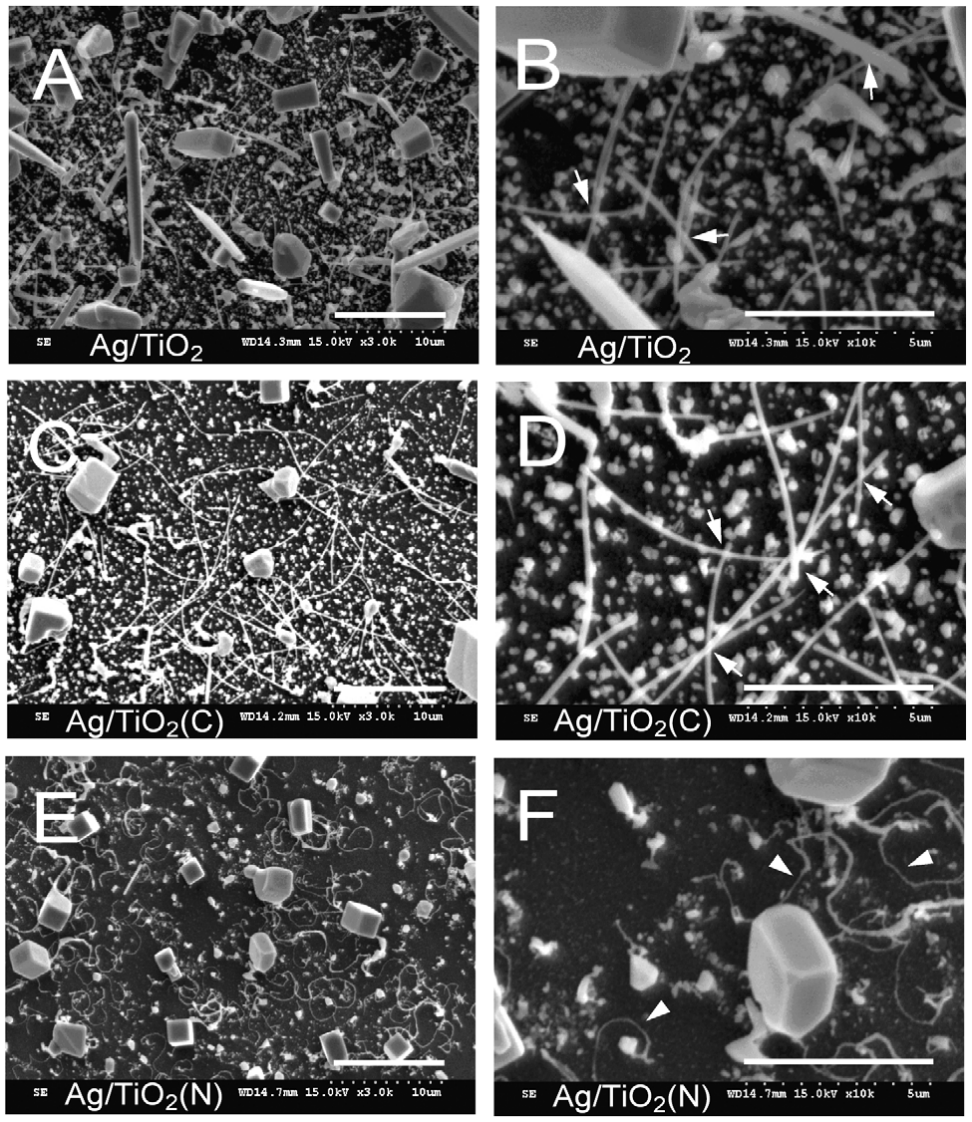
Scanning electron microscope images of Ag/TiO_2_ substrates. TiO_2_ (A, B), TiO_2_ (C) (C, D) and TiO_2_ (N) (E, F) substrates were coated with silver (thermal reduced from 0.1 M AgNO_3_). Images with both low (A, C, E) and high (B, D, F) magnifications were shown. Arrows indicate the crossings of the rod-shaped silver nanoparticles (B, D); arrow heads indicate the wire-like silver nanoparticles (F). Scale bar: 10 µm (A, C, E), 5 µm (B, D, F).

We used scanning electron microscope (SEM) to investigate whether there are morphological differences of Ag-depositions on TiO_2_, TiO_2_ (C) and TiO_2_ (N) substrates (0.1 M AgNO_3_ groups in Fig. 2). The SEM images indicated that the amount of crystalline silver on TiO_2_ (N) surfaces was not significantly higher then those on TiO_2_ and TiO_2_ (C) groups (Fig. 4, E vs. A, C and F vs. B, D). Thus, the better performance of Ag/TiO_2_ (N) is not attributed to higher amount of bactericidal silver-coating. Intriguingly, besides micrometer-scale crystals with rectangular parallelepiped shapes, all three groups showed deposition of fibrous silver nanoparticles on their surfaces (Fig. 4A–F). In addition, we found unique wire-like crystalline silver on TiO_2_ (N) groups (Fig. 4, E, F vs. A–D; arrow heads in F). When compared with fibrous rod-shaped crystalline silver on Ag/TiO_2_ and Ag/TiO_2_ (C) groups with frequently observed crossings (Fig. 4B, D, arrows), these wire-like crystalline silver on TiO_2_ (N) surfaces seem to be adhered relatively closer to TiO_2_ surfaces without obvious crossing fibers (Fig. 4E, F).

### Bactericidal activities of Ag/TiO_2_ on the elimination of pathogens

**Figure 5:**
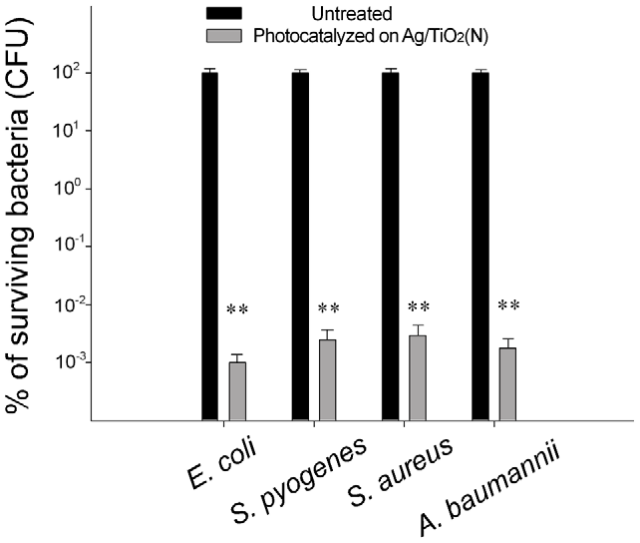
Pathogen analysis. Photocatalysis-mediated antibacterial activity of Ag/TiO_2_ (N) substrates by visible-light illumination was evaluated using nonpathogenic *E. coli*, and pathogenic bacteria including *S. pyogenes, S. aureus*, and *A. baumannii*. The silver coating on TiO_2_ (N) substrates was thermal reduced from 0.1 M AgNO_3_. For each pathogen, the viable bacterial number of initial inputs (untreated groups, 1×10^7^ CFU) was normalized to 100%. ** *P*<0.01 compared to the respective untreated groups.

To further investigate the performance of Ag/TiO_2_ (N) to eradicate pathogenic bacteria, human pathogens including *S. pyogenes, S. aureus* and *A. baumannii* were subjected to visible-light induced catalysis on Ag/TiO_2_ (N) substrates (0.1 M AgNO_3_ groups). We found that all tested pathogens were significantly eliminated. The effectiveness showed an approximately 5 log reduction of bacterial population, which was not influenced by whether the target was Gram-positive or Gram-negative bacteria (Fig. 5).

## Discussion

Antimicrobial property of silver has been used to treat burns and wounds for thousands of year [28]. Compared to silver, photocatalyst-based disinfectants are still under developmental stage. Although the bactericidal activity is relatively less than silver products, chemically inert TiO_2_ materials have the advantages to exert its bactericidal activity persistently and to be modulating by light that makes it potentially to be applied on different usages. Besides the advantage of antibacterial property, however, there are still unavoidable side effects of both silver and TiO_2_ products. Exposure to soluble silver compounds may elicit toxic effects on respiratory and circulation systems, and organ damages on eye, skin, liver and kidney [29]. Chronic effects are involving permanent bluish-grey discoloration of the skin (argyria) and eyes (argyrosis) [29]. Currently there is no report referring to the toxic effect of TiO_2_ thin films. TiO_2_ nanoparticles were shown have no toxic effect after ingested by animals [30]. Ultrafine-TiO_2_ particles, however, induced pulmonary toxicity in rats [31]. Combined treatments would reduce the affective dosage of both agents and may thus reduce the side effects. Synergistic and enhanced effects were shown in the combined treatments of silver with other antibacterial agents like UV-irradiation and antibiotics [32]–[36]. Studies using silver/TiO_2_ combination were also indicated. Despite the high antibacterial activity of silver alone that consistently appeared in all relevant literatures, the photocatalytic destruction of *E. coli* was enhanced in some [22]–[25], but not all conditions [26]. This indicates that a re-justification of silver coating is needed. As a result, here we choose thin films, a non-toxic form of TiO_2_ to establish the correlation of thermo-reduction/substrate conditions with antibacterial performance. In addition to commonly used bacterium *E. coli*, we also tested relatively rarely discussed pathogens. Here we demonstrated the first time that silver and visible-responsive photocatalyst substrates TiO_2_ (N) synergistically eliminated pathogens under visible-light illumination. Previously we found that illuminating TiO_2_ (N) substrates could only result in less than one log inhibition of bacterial population [13], [17]; while in this study, TiO_2_ (N) substrates caused two log reduction in companion with silver (Fig. 3 Light vs. Dark in 5 and 10 min groups). Since TiO_2_ (N)-mediated photocatalysis could injure the bacterial spores and made them more vulnerable to host immune system [17], such injuries might also lead to a higher sensitivity of bacteria in responding to silver treatments. Detail mechanism of this synergistic effect is worthy to be further investigated.

Here we found the superior antibacterial activity is associated with the formation of wire-like silver nanoparticles. Size and shape were shown to influence the bactericidal property of silver nanoparticles [37], [38]. Basically, the smaller size they are, the greater their surface area to volume ratio and the higher microbial contacting efficiency. Evidence indicates the silver nanoparticles interacting with human immunodeficiency virus (HIV)-1 in a size dependent manner [39]; and silver particles smaller than 10 nm have a preferentially higher bactericidal activity as compared to larger particles up to 100 nm [37]. Experiments using silver nanoparticles with different shapes further indicated that truncated triangular nanoparticles got a best antibacterial performance with an affective dose at 0.1 µg/L, as compared with spherical- and rod-shape particles with affective doses at 1.25 µg/L and 5–10 µg/L, respectively [38]. These results suggested that both size and shape are critical factors to determine the antibacterial property of silver nanoparticles. As a result, it is reasonable that wire-shaped silver nanoparticles/TiO_2_ (N) substrates exhibit a stronger bactericidal activity. Specific role of wire-like structure contribute to the antibacterial effect of silver remains to be further investigated.

Taken together, here we successfully demonstrated that the antibacterial property of silver could be optimized through controlling the thermo-reducing conditions of silver on titania substrates. The silver and TiO_2_ (N) composites showed a synergistic antibacterial activity under visible light illumination. These findings suggest that silver/TiO_2_ (N) composited material has potential application in the development of alternative disinfectants.

## Materials and Methods

### Preparation of Ag-coated TiO_2_, TiO_2_ (C) and TiO_2_ (N) substrates

The substrates used to coat silver nanostructures were three types of titania films, pure TiO_2_, carbon doped TiO_2_ (C) and nitrogen-doped TiO_2_ (N) deposited on Si or quartz coupons using an ion-assisted electron-beam evaporation system (Branchy Vacuum Technology Co., Ltd., Taoyuan, Taiwan) as described [13]. The base pressure of the deposition chamber was below 2.7×10^−4^ Pa. The substrates were sputter-etched with argon ions (Ar^+^) for 5 minutes prior to the deposition to remove any residual pollutants on the surface. The substrate temperature was maintained at 300°C by quartz lamps. The TiO_2_ films were deposited in oxygen atmosphere (6.7×10^−3^ Pa) using rutile TiO_2_ (99.99%) as a source material. The doped titania films were prepared in a similar manner as pure titania except the bombardment of energetic dopant ions on the growing film through the ion gun. The nitrogen flow for TiO_2−x_N_x_ films was 15 standard cm^3^ min^−1^ through the ion gun and the chamber pressure was at 4.4×10^−2^ Pa. The carbon dioxide gas flow for TiO_2−x_C_x_ films was 7 standard cm^3^ min^−1^, and the chamber pressure was 2.6×10^−2^ Pa. The ion gun beam current of 10 mA and voltage of −1000 V were maintained by a Commonwealth Scientific ion beam power supply controller. The three films were prepared under the optimized conditions for their categories of anatase crystallinity and dopant concentration [40]–[42].

In the preparation of silver nanostructures by thermal reduction method, the titania film coated substrates were placed in 1 M, 0.1 M and 0.0125 M aqueous solutions of silver nitrate, AgNO_3_ (Sigma-Aldrich Corp., USA), and then pulled out. Thus, a layer of silver nitrate solution was absorbed on substrate surface. These substrates with silver nitrate were placed in an aluminum oxide crucible and heat-treated in a furnace in air. The heat-treatment temperature was raised at a rate of 5°C per minute to 200°C, maintained for 20 minutes, and then raised again at the same rate to 300°C. They were finally furnace-cooled after being held at 300°C for 3 hours.

### Bacterial strains and culture

*E. coli* (strain OP50) was maintained and cultured Luria-Bertani broth or Luria-Bertani broth agar (MDBio, Inc. Taipei, Taiwan) as previously described [43], [44]. *Streptococcus pyogenes* (strain M29588) and *S. aureus* (strain SA02) (without antibiotic resistance) and pan-drug resistance *A. baumannii* (PDRAB; strain M36788), are clinical isolates from the Buddhist Tzu-Chi General Hospital, Hualien, Taiwan. *S. pyogenes*, *S. aureus* were grown in tryptic soy broth supplemented with 0.5% yeast extract (TSBY) broth or TSBY broth agar (MDBio, Inc. Taipei, Taiwan) [13], [45].

### Bacterial killing experiments

In this study, bacterial concentrations were either determined by the standard plating method or inferred from optical density readings at 600 nm (OD_600_). For each bacterium, a factor for converting the OD_600_ values of the bacterial culture to concentration (CFU/ml) was calculated as the followings. A fresh bacterial culture was diluted by factors of 10^−1^ to 10^−7^, and OD_600_ of these dilutions was measured. Bacterial concentrations of these dilutions were determined using standard plating method. The OD_600_ values were plotted against the bacterial concentrations' log values, and the conversion factors for particular bacteria were calculated. The conversion factor for *S. aureus*, for example, was calculated to be 1×10^8^ CFU/ml per OD_600_ by this method.

In order to determine the bactericidal effects of the silver-coated titania films, 200 µl of bacterial overnight culture was transferred into 5 ml of culture medium and incubated at 37°C until an OD_600_ of 0.3 to 0.6 (log phase) was reached. The bacterial concentrations were calculated using the conversion factor for the bacteria, and the cultures were diluted to 1×10^8^ CFU/ml with culture medium. One hundred microliters of the bacterial culture (1×10^7^ CFU) was then applied to an area of approximately 2 cm^2^ of the different substrates using a plastic yellow tip. Before loaded with bacterial solution, the Ag/TiO_2_ surfaces were gently wished with distilled water for three times. The bacteria substrates were then placed under an incandescent lamp (Classictone incandescent lamp, 60 W, Philips, Taiwan) for photocatalytic reaction, and a light meter (model LX-102, Lutron Electronic Enterprises, Taiwan) was used to record the illumination density. The photocatalytic reaction (bacterial killing) was carried out in a 4°C cold room to eliminate the effect of heat. In the initial experiments, illuminations were carried out for 10 min at a distance of 10 cm, corresponding to the illumination density of 1.2×10^3^ lumen/m^2^ (lux) (30 mW/cm^2^). In the kinetic analysis experiments, illuminations were carried out for 1, 5, and 10 min at a distance of 10 cm, and illumination density of 1.2×10^3^ lux (30 mW/cm^2^). After illumination, the bacterial solutions were recovered from the titania substrates, and an aliquot of fresh culture medium (250 µl) was used to flush the wells through repeatedly pipetting to further collect the residual bacteria on the wells of the culture dish. The two bacterial solutions were pooled to make a total of 350 µl. The bacterial concentration was determined by the standard plating method immediately after the bacterial collection, and percentage of surviving bacteria was calculated.

### Scanning electron microscopic imaging

Scanning electron microscopic (SEM) images were obtained using a JEM6500-F field-emission scanning electron microscopy (FESEM) (JEOL Ltd., Tokyo, Japan).

### Statistical analysis

All results were calculated from data of three independent experiments. *T*-test was used to assess statistical significance of differences in results of the antimicrobial effects. A *P* value of less than 0.05 (*P*<0.05) was considered significant. The statistical tests were carried out and output to graphs using the Microsoft Excel (Microsoft Taiwan, Taipei, Taiwan) and SigmaPlot (Systat Software, Point Richmond, CA, USA) software.
